# Determination of factors associated with serum cholesterol response to dairy fat consumption in overweight adults: Secondary analysis from an RCT

**DOI:** 10.3389/fnut.2022.945723

**Published:** 2022-08-03

**Authors:** Aileen O'Connor, Emma L. Feeney, Nupur Bhargava, Nessa Noronha, Eileen R. Gibney

**Affiliations:** ^1^School of Agriculture and Food Science, Institute of Food and Health, University College Dublin, Dublin, Ireland; ^2^Food for Health Ireland, Dublin, Ireland

**Keywords:** cheese, cholesterol, dairy matrix, saturated fat, response

## Abstract

Elevated intakes of saturated fatty acids (SFA) can adversely affect serum cholesterol levels. Dairy fat contains ~60% SFA, prompting healthy eating guidelines to recommend low-fat dairy. Physiological, and environmental factors influence inter-individual variance in response to food consumption. Evidence exploring the dairy matrix has differing effects of dairy fat consumption on serum cholesterol levels when consumed in the form of cheese. The extent of this variability and determinants of response to dairy fat are currently unknown. The objective of this study was to determine factors associated with lipid metabolism response to a dairy fat intervention, with a focus on serum cholesterol. A 6-week randomized parallel intervention trial was carried out in healthy volunteers (≥50 years, BMI ≥25 kg/m^2^). Participants (*n* = 104) consumed ~40 g dairy fat daily in addition to their usual diet, in 1 of 3 forms: butter, cheese, or reduced-fat cheese and butter. For this analysis, “response” was based on the percentage (%) change in serum total cholesterol (TC), low-density lipoprotein cholesterol (LDL-c), and high-density lipoprotein cholesterol (HDL-c) from pre- to post-intervention. Participants were divided into tertiles for each lipid response. The upper and lower tertiles were used to categorize participants as “responders” and “non-responders.” For TC and LDL-c, response was classified as a decrease, whereas “response” was defined as an increase for HDL-c. Clinical response was also considered, by calculating pre- and post-intervention prevalence of those meeting target levels of cholesterol recommendations. Participants demonstrating the largest % decrease (Tertile 1; “responders”) in TC had significantly higher levels of TC and HDL-c, at baseline, and lower levels of triglycerides (TAGs) compared to those in tertile 3 (i.e., TC non-responders). Those with the largest % decrease in LDL-c (Tertile 1: LDL-c responders) had higher baseline levels of LDL-c and lower levels of TAGs. Multiple regression analysis revealed that the % change in TC and LDL-c was associated with baseline TC, TAG, body weight and high-sensitivity C-reactive protein (hsCRP; *P* < 0.05). Previous work has demonstrated the dairy food matrix affects lipid response to dairy consumption. This study suggests that phenotypic differences may also influence response to dairy fat in overweight individuals.

## Introduction

Inter-individual variance in response to consumption of food or nutrients is influenced by a variety of physiological and environmental factors, which can impact the link between diet and an individuals' risk of various related diseases (1, 2). Even in the controlled environment of nutrition research, individuals' responses to dietary interventions are shown to be highly variable (3–5). It is important to identify and understand these variances, not only as they can affect our interpretation of the results, but they can also support the development of tailored nutrition advice and delivery of personalized nutrition (6). Tailoring nutrition or dietary advice to an individual based on their needs and requirements offers precision nutrition which can improve overall health as well as reducing the risk of diet-related diseases (7, 8).

Several studies have examined inter-individual variability in response to lipid consumption, noting influences from phenotypic and genetic factors (9–13). For example, using data from the MECHE (Metabolic Challenge) study, Ryan et al. (9) examined baseline characteristics influencing response to standard oral lipid tolerance tests (OLTT). Influencing factors included age, triacylglycerols (TAGs), circulating fatty acids, and several single-nucleotide polymorphisms (SNP) (9). Using the same data, Morris et al. (10) found that fitness levels also influenced response following lipid consumption. Furthermore, some studies have reported sex differences in response to lipid consumption, such as alpha-linoleic acid enriched margarines and spreads (11).

Whilst evidence of factors influencing variation to interventions is growing, the complexity within nutrition research is, in fact, 2-fold. One must consider the impact of the food matrix and the source of the nutrients within the intervention, as well as the individuals' characteristics influencing response. A systematic review and meta-analysis conducted by de Souza et al. (14), re-opened the debate on intake of saturated fatty acids (SFA), where associations between intake of total fat, SFA and *trans*-unsaturated fat with all-cause mortality and differing morbidities were examined. Contrary to previous evidence, they reported that SFA intake was not associated with all-cause mortality, as well as several non-communicable diseases such as cardiovascular disease (CVD), total coronary heart disease (CHD), ischemic stroke, and type 2 diabetes (14). While the totality of evidence indicates that elevated levels of SFA intake is adversely associated with non-communicable disease risk, a number of meta-analyses have suggested that not all total SFA is associated with such (14–16). This supports the concept that the specific type of SFA as well as the variation of fat in food can play a significant role in determining this risk. Dairy foods are naturally rich sources of a wide range of nutrients, including protein, fats, and several micronutrients (17). Most dairy fat typically contains high amounts of SFA (~60%), thus it has been targeted as one of the main causes of diet-related diseases such as CVD (18). Approximately 20% of SFA intakes across Europe and the United States are from dairy products, prompting many healthy eating guidelines to recommend low-fat dairy (19). However, focusing specifically on fat derived from the dairy food group, some meta-analyses have reported that when SFA is consumed within the dairy matrix, it may have favorable or neutral associations with cardiometabolic health (20, 21). Moreover, some studies have observed contrasting risks associated with different dairy products (22). Much like the understanding of factors influencing response to lipid intakes, more evidence is needed to understand factors influencing response to dairy fat consumption across the different food matrices. Considering these findings, the most recent Scientific Advisory Committee on Nutrition (SACN) report (23) concluded that the totality of evidence did not provide a basis to amend the current SFA recommendations which remains at, <10% total energy (23).

Recently, we presented evidence with respect to the consumption of dairy fat within and outside of, the cheese matrix (24). A significant reduction in triglycerides (TAGs) and low-density lipoprotein cholesterol (LDL-c) was observed post-intervention when the fat was contained within the cheese matrix, compared to butter, and reduced-fat cheese matrices (24). However, within that study, considerable variation in response to the consumption of dairy fat, in all forms, was evident. Understanding the factors influencing the response to dairy fat, within and outside the cheese matrix, is an important step in order to contribute to tailored and specific advice with respect to dairy. Therefore, the objective of this study was firstly, to identify the responders and non-responders of this dairy fat intervention study; secondly, to determine the factors influencing lipid metabolism response, and thirdly, to investigate the relationship between them.

## Methods

### Participants

In the original study, a total of 203 participants were recruited from Dublin, Ireland, and the surrounding areas. The study inclusion criteria were as follows: healthy volunteers aged ≥50 y and a BMI ≥25 kg/m^2^. Exclusion criteria were as follows: any prescribed medication for cholesterol or blood pressure lowering reasons, any prescribed diet, or actively trying to lose weight. Of the 203 participants randomized to the study, 164 participants completed the study. Written, informed consent was obtained from all participants before commencing the study, and all procedures involving the study participants were approved by the Human Research Ethics Committee of University College Dublin (LS-15-44-Feeney-Gibney). Full details are available elsewhere (24).

### Study design

In brief, the study was a randomized parallel controlled trial divided into four arms: (A) full-fat cheddar cheese; (B) reduced-fat cheese plus butter; and (C) butter, calcium caseinate powder, and a calcium supplement (CaCO_3_). Group D followed the same intervention diet as Group A, but they completed a run-in period prior to starting the intervention, abstaining from all cheese for 6 weeks. All intervention diets delivered between 39 and 41 g/d (41 g ± 1.6 g) of dairy fat in different dairy matrices over a 6-week period. The intervention diets were also matched as closely as possible for total energy, macronutrients, and calcium. More information on this nutrient breakdown can be found in Supplementary Table 1. For this analysis, group D was excluded as it was the only group to complete a run-in period of no dietary cheese. The current analysis was performed on data from participants who reported 80% compliance or greater (*per protocol*) (*n* = 104). Anthropometry measurements were collected at baseline and post-intervention. Fasting body weight was measured using a Tanita scale, Model BC-420 ma, and height was measured with a free standing SECA stadiometer. Serum and plasma fasting blood samples were also collected at baseline and post-intervention. For a more detailed description of this protocol see Feeney et al. (24).

Change in LDL-c was the primary outcome from baseline to post-intervention between Group A and C (24). The change in overall lipid profile [total cholesterol (TC), LDL-c and high-density lipoprotein cholesterol (HDL-c)] and several metabolic markers such as TAGs, non-esterified fatty acids (NEFA), glucose, insulin, high-sensitivity C-reactive protein (hsCRP), and blood pressure (BP) were examined (24). Further information on the study outcomes can be found in the original paper (24).

### Defining responders

The aim of this secondary analysis was to determine factors associated with individual lipid metabolism response to dairy fat consumption, outside of the dairy food matrix in which they were presented. “Response” was based on the percentage (%) change (Δ) in serum TC, LDL-c, and HDL-c from pre- to post-intervention. Participants were ranked from the largest decrease to the largest increase for each of these lipid parameters. The ranked participants were divided into tertiles (three groups of equal size) for each lipid response; TC, LDL-c, and HDL-c. The tertiles were used to categorize participants as “responders” and “non-responders” at a total cohort level, irrespective of intervention group. This was also conducted for each intervention group but as results were similar, we combined them as a single overall dairy fat intervention here to understand response; see Supplementary Tables 2A–C. Supplementary Figures 1–3 also show similar patterns of variation in response across each group. For TC and LDL-c, tertile 1 demonstrated the largest % decrease and was classified as a response, whereas tertile 3 displayed the largest % increase and was classified as a non-response. The opposite applied across tertiles of HDL-c, where participants within tertile 3 (largest % increase) were classified as a response and those within tertile 1 were considered a non-response. The identified responders and non-responders were then compared across each lipid parameter; serum TC, LDL-c, and HDL-c.

Baseline characteristics considered included circulating serum cholesterol, TAGs, NEFA, inflammatory marker hsCRP, glucose, insulin, and BP, as well as several anthropometric measurements. To examine the clinical relevance of the effects of this dairy fat intervention, participants were also categorized based on clinical risk categories of circulating cholesterol levels, i.e., above, or below recommended lipid levels. The cut offs, adopted from WHO (25), were applied for TC, LDL-c, and HDL-c.

### Statistical analysis

Statistical analyses were conducted using SPSS^®^ V24.0 for Windows™ (SPSS Inc. Chicago, IL, USA). Descriptive statistics were performed on baseline data which included demographic, anthropometric, and clinical chemistry data for the total population and stratified by diet intervention. Sex was reported as a percentage. Delta (Δ) scores (changes) were calculated by subtracting the post-intervention values from the pre-intervention values. Multivariate analysis was used to evaluate differences in baseline characteristics across the tertiles while adjusting for sex and for study wave, as per previous analysis (24). Study wave refers to each separate batch of cheese issued to participants over the total study duration. While all cheese used within each study wave was at the same stage of ripening to ensure continuity, seasonal variation in milk composition can impact the composition of the cheese, and as such was included within the analysis. A multiple regression model was generated to examine the baseline variables predictive of % change for the lipid parameters. With respect to clinical relevance, the proportion of participants meeting the recommended lipid levels between baseline and post-intervention were examined by means of a chi-squared test. The Bonferroni correction for multiple comparisons was applied, and significance level for all statistical analyses was classified as *P* ≤ 0.05. Analysis was considered in the total compliant cohort and within intervention arm, as appropriate.

## Results

### Distribution of individual responses

Table 1 displays baseline characteristics across the intervention groups. Of these, only age differed across the intervention groups, whereby group A was significantly older than group B (63 vs. 58 years, respectively; *P* = 0.009).

**Table 1:** Baseline demographic and phenotypic characteristics of the total population and by intervention groups A–C.

	**Total population**		**Group A**		**Group B**		**Group C**		
	***n*** **104**		***n*** **40**		***n*** **36**		***n*** **28**		
	**Mean**	**SD**	**Mean**	**SD**	**Mean**	**SD**	**Mean**	**SD**	*P* ^*^
**Gender (%)**
Male	41		40		36		50		0.522
Female	59		60		64		50		
**Baseline characteristics**
Age (years)	60.4	6.9	62.9^a^	6.7	58.4^b^	6.0	59.2^ab^	7.4	0.009
Weight (kg)	78.6	13.7	78.8	13.9	76.9	13.0	80.6	14.4	0.553
BMI (kg/m^2^)	27.6	3.6	27.7	2.7	27.3	3.9	27.9	4.2	0.778
Body fat (%)	33.8	7.6	34.4	7.9	33.8	7.0	33.0	7.9	0.762
Systolic BP (mmHg)^¥^	130.1	18.7	131.4	15.8	130.7	22.8	127.3	17.0	0.659
Diastolic BP (mmHg)^†^	83.1	12.4	82.5	12.5	85.1	12.8	81.3	11.7	0.466
Total cholesterol (mmol/l)	5.89	0.97	5.75	1.09	6.18	0.98	5.72	0.70	0.089
HDL cholesterol (mmol/l)	1.70	0.49	1.73	0.49	1.79	0.57	1.56	0.33	0.080
LDL cholesterol (mmol/l)	3.61	0.84	3.42	0.87	3.85	0.89	3.57	0.66	0.077
TAGs (mmol/l)	1.27	0.55	1.32	0.57	1.19	0.47	1.30	0.61	0.527
NEFA (mmol/l)	0.64	0.32	0.63	0.30	0.68	0.36	0.62	0.29	0.714
Glucose (mmol/l)	6.17	0.61	6.15	0.52	6.14	0.58	6.23	0.77	0.802
Insulin (mU/L)	5.71	2.88	6.00	2.89	5.51	2.56	5.56	3.31	0.727
hsCRP (mg/L)	2.41	2.42	2.52	2.98	2.62	2.08	1.97	1.91	0.539

* *One-way ANOVA was used with Bonferroni post hoc test and chi-squared test to assess baseline differences between intervention groups A–C (P < 0.05)*.

¥ *excludes 3 missing values;*

† *excludes 3 missing values*.

ab *Different superscript letters indicate significant differences in mean values across the groups (P < 0.05). n, number; BMI, body mass index; BP, blood pressure; LDL, low-density lipoprotein; HDL, high-density lipoprotein; TAGs, triacylglycerols; NEFA, non-esterified fatty acids; hsCRP, high-sensitivity C-reactive protein*.

### Changes in lipid biomarkers

**Figure 1:**
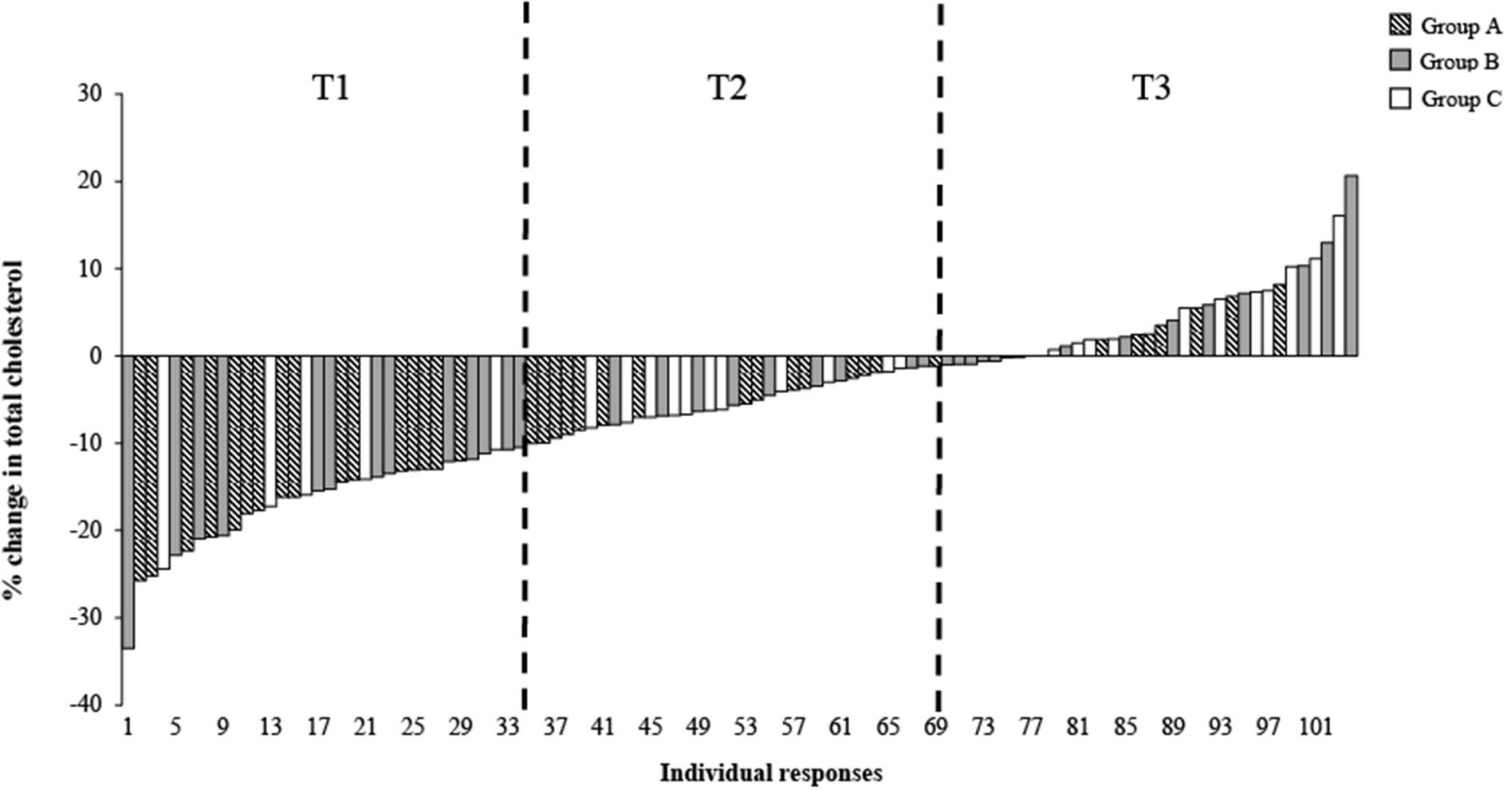
Distribution of individual responses for total cholesterol (%) following 6 weeks of a dairy fat intervention across the total population and the three diet intervention groups. Each bar represents an individual's % total cholesterol change Δ, delta; T1, Tertile 1; T2, Tertile 2; T3, Tertile 3.

Figure 1 illustrates the individual responses for the % change in TC after 6 weeks of a dairy fat intervention across the total population, categorized by intervention groups A-C, with the tertiles of TC % change indicated. The plotted individual % changes were sorted by the magnitude of change, ranging in order from the largest decrease to the largest increase. As expected, there was a significant difference in the % change of TC across the tertiles (total population) (Table 2). The mean % change across tertiles of TC were split as follows: −16.8% ± 5.3% (responder), −5.3% ± 2.7% and 4.6% ± 5.2% (non-responder; *P* < 0.001). When the tertiles were further stratified by intervention group, the same trends were evident between the groups across the tertiles (*P* < 0.001). However, when the % change was split by intervention group within each tertile, all groups demonstrated similar changes in TC cholesterol levels and were no longer significant (Table 2).

**Table 2:** Percentage change in TC, LDL-c, and HDL-c across tertiles of percentage change at total population level, compared across intervention groups A–C.

	**T1**			**T2**			**T3**			*P* ^*^
	***n***	**Mean**	**SD**	***n***	**Mean**	**SD**	***n***	**Mean**	**SD**	
**Total cholesterol** Δ **(%)**										
Total population	34	−16.76^a^	5.31	35	−5.35^b^	2.73	35	4.59^c^	5.21	<0.001
Group A	16	−17.18^a^	4.47	15	−5.86^b^	3.17	9	3.39^c^	2.91	<0.001
Group B	13	−16.33^a^	6.60	9	−4.47^b^	2.40	14	4.28^c^	6.54	<0.001
Group C	5	−16.50^a^	5.06	11	−5.38^b^	2.38	12	5.85^c^	4.93	<0.001
*P* ^¥^		0.834			0.676			0.699		
**LDL-c** **Δ** **(%)**										
Total population	34	−22.41^a^	5.48	35	−8.70^b^	2.88	35	7.67^c^	11.27	<0.001
Group A	18	−22.29^a^	5.49	13	−8.77^b^	3.01	9	4.97^c^	7.97	<0.001
Group B	10	−23.31^a^	6.53	12	−8.17^b^	3.08	14	7.23^c^	11.57	<0.001
Group C	6	−21.29^a^	4.03	10	−9.23^b^	2.61	12	10.21^c^	13.24	<0.001
*P* ^¥^		0.890			0.645			0.824		
**HDL-c** **Δ** **(%)**										
Total population	34	−12.41^a^	6.40	36	−0.47^b^	3.01	34	14.33^c^	9.71	<0.001
Group A	7	−15.02^a^	7.90	21	−0.31^b^	2.96	12	11.74^c^	6.48	<0.001
Group B	16	−12.77^a^	7.00	10	−0.30^b^	2.81	10	11.49^c^	4.23	<0.001
Group C	11	−10.21^a^	3.74	5	−1.46^b^	4.01	12	19.29^c^	13.60	<0.001
*P* ^¥^		0.395			0.773			0.130		

* *Univariate analysis of baseline characteristics to assess differences between tertiles of percentage change, with gender and study wave as covariates (P < 0.05)*.

¥ *Univariate analysis of baseline characteristics to assess differences between intervention groups A–C, with gender and study wave as covariates (P < 0.05). Both used Bonferroni correction method for multiple comparisons*.

abc *Different superscript letters indicate significant differences in mean values across tertiles. n, number; SD, standard deviation; Δ, delta; T1, Tertile 1; T2, Tertile 2; T3, Tertile 3; LDL-c, low-density lipoprotein; HDL-c, high-density lipoprotein*.

Similar trends to the above were observed when the % change of LDL-c and HDL-c were examined across tertiles of LDL-c and HDL-c % change, respectively. As expected, significant differences across tertiles of LDL-c % change at a total population level were apparent, with tertiles 1-3 displaying the following: T1, −22.4 ± 5.5% (responder); T2, −8.8 ± 2.8% and T3, 7.4 ± 11.5% (non-responder; *P* < 0.001).

**Figure 2:**
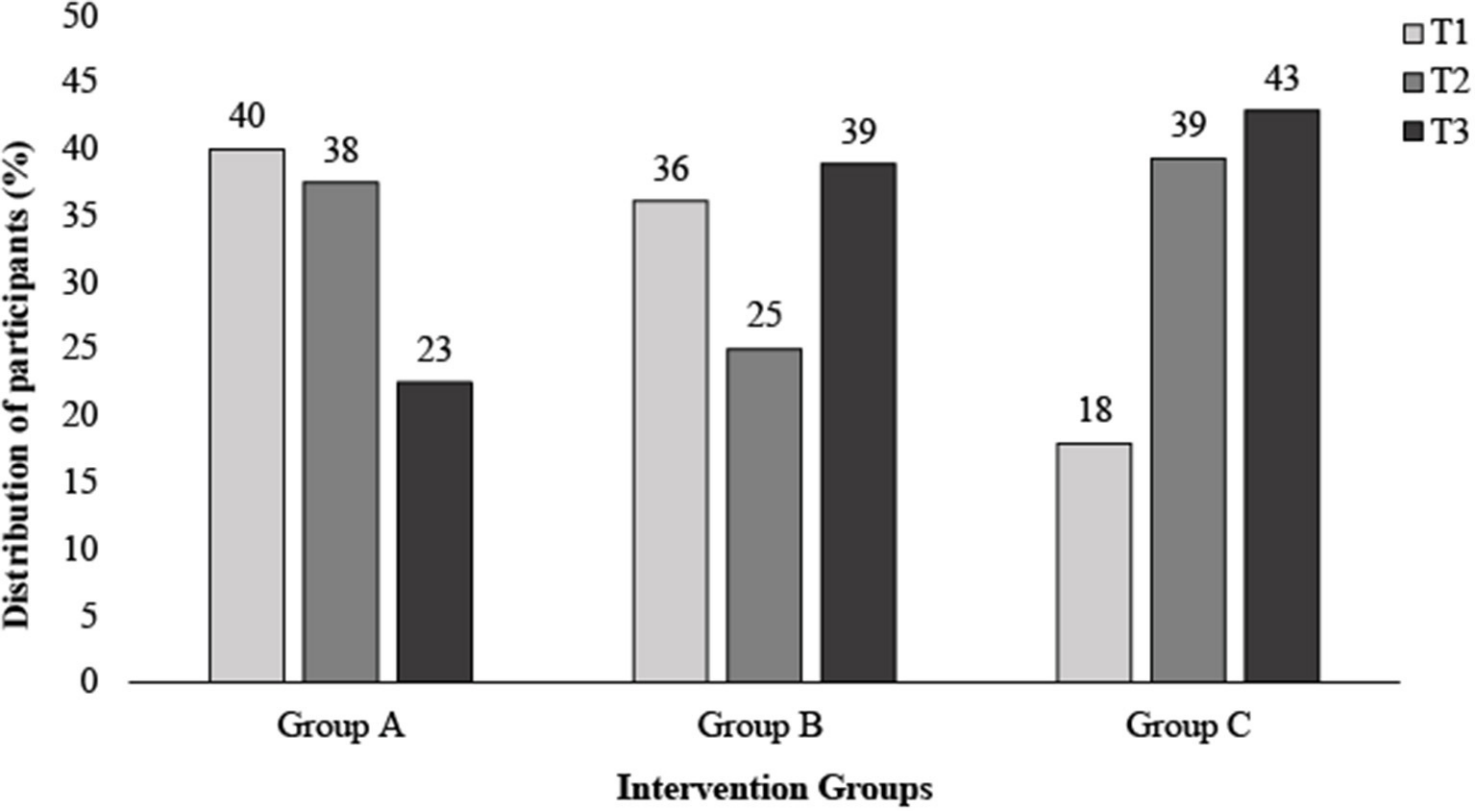
Distribution of participants across tertiles of total cholesterol Δ (%) following 6 weeks of a dairy fat intervention split by diet intervention group. Δ, delta; T1, Tertile 1; T2, Tertile 2; T3, Tertile 3.

Similarly, differences across tertiles of HDL-c % change was observed, with tertiles 1-3 displaying the following: T1, −12.41 ± 6.4% (non-responder); T2, −0.47 ± 3.01% and T3, 14.33 ± 9.71% (responder; *P* < 0.001). No significant differences were observed across groups within tertiles for both LDL-c and HDL-c % change (*P* < 0.05) (Table 2). Additional analysis carried out on the % change in cholesterol across tertiles of % change within each intervention group can be seen in Supplementary Table 3, with some significant differences noted between groups. Examining differences in distribution within tertiles of % change of TC, Figure 2 demonstrates a greater proportion of individuals within T1 in group A (40%), compared to group C (18%) (Figure 2).

### Baseline anthropometric and metabolic characteristics across tertiles of response

Tables 3A,B present baseline characteristics across tertiles of % change in TC and LDL-c levels for the total population, respectively. Tertile analysis was also carried out for HDL-c (Supplementary Table 4). As no changes were apparent in HDL-c between groups in our previous study (24), the remaining results will focus on identifying factors influencing TC and LDL-c response. No demographic or anthropometric differences were apparent across TC and LDL-c tertiles. Participants showing the largest % decrease (responders) in TC displayed significantly higher levels of TC and HDL-c, and lower levels of TAGs at baseline (6.24 ± 1.15; 1.90 ± 0.50; 1.08 ± 0.39 mmol/l, respectively) compared to those displaying the largest % increase (non-responders) (5.54 ± 0.77, 1.49 ± 0.34 and 1.43 ± 0.66 mmol/l, respectively). Across tertiles of LDL-c % change, those with the largest % decrease (responders) showed similar significant differences, reporting higher baseline levels of LDL-c and TAG (3.90 ± 0.91; 1.07 ± 0.41 mmol/l, respectively) compared to T2 and T3. When categorized by HDL-c % change, TC was significantly higher in those with the largest decreases compared to T2 and T3 (*P* < 0.018; Supplementary Table 3). This was similar for HDL-c (*P* < 0.001). While many of the baseline characteristics remained non-significant, similar trends for TC, LDL-c, HDL-c, and TAGs were observed when split by group (data not shown).

**Table 3A:** Comparison of baseline characteristics across tertiles of percentage change in circulating serum total cholesterol levels for total population.

	**Total Cholesterol**						
	**Tertile 1**		**Tertile 2**		**Tertile 3**		
	***n*** **34**		***n*** **35**		***n*** **35**		
	**Mean**	**SD**	**Mean**	**SD**	**Mean**	**SD**	P^*^
Total cholesterol Δ (%)	−16.76^a^	5.31	−5.28^b^	2.74	4.18^c^	4.95	<0.001
**Gender (%)**
Male	38		37		49		0.564^¥^
Female	62		63		51		
**Baseline characteristics**
Age (years)	61.1	7.1	60.2	7.5	59.7	6.0	0.747
Weight (kg)	79.8	11.2	79.7	16.5	76.0	13.2	0.105
BMI (kg/m^2^)	27.8	3.6	27.9	3.7	26.9	3.6	0.415
Body fat (%)	34.1	8.4	34.7	6.8	32.3	7.6	0.614
Systolic BP (mmHg)^§^	126.7	16.2	133.1	20.8	130.5	18.8	0.360
Diastolic BP (mmHg)^†^	80.3	10.4	86.1	12.9	82.8	13.2	0.135
Total cholesterol (mmol/l)	6.24^a^	1.15	5.85^a^^b^	0.82	5.54^b^	0.77	0.018
HDL-cholesterol (mmol/l)	1.90^a^	0.50	1.73^a^^b^	0.54	1.49^b^	0.34	0.003
LDL-cholesterol (mmol/l)	3.85	0.99	3.54	0.69	3.40	0.73	0.086
TAGs (mmol/l)	1.08^a^	0.39	1.28^a^^b^	0.54	1.43^b^	0.66	0.043
NEFA (mmol/l)	0.62	0.32	0.68	0.29	0.63	0.35	0.724
Glucose (mmol/l)	6.13	0.51	6.25	0.72	6.10	0.60	0.464
Insulin (mU/L)	5.11	2.28	5.69	2.95	6.20	3.38	0.398
hsCRP (mg/L)	2.09	1.91	2.11	1.56	2.98	3.42	0.169

§ *Excludes 3 missing values;*

† *excludes 3 missing values*.

* *Multivariate analysis of baseline characteristics to assess difference between tertiles of responders, with gender and study wave as covariates (P < 0.05), using Bonferroni correction method for multiple comparisons*.

¥ *Chi-squared test was used to assess differences in gender across the tertiles (P < 0.05)*.

abc *Different superscript letters indicate significant differences in mean values across tertiles. n, number; SD, standard deviation; Δ, delta; T1, Tertile 1; T2, Tertile 2; T3, Tertile 3; LDL, low-density lipoprotein; HDL, high-density lipoprotein. BMI, body mass index; BP, blood pressure; LDL, low-density lipoprotein; HDL, high-density lipoprotein; TAGs, triacylglycerols; NEFA, non-esterified fatty acids; hsCRP, high-sensitivity C-reactive protein*.

**Table 3B:** Comparison of baseline characteristics across tertiles of percentage change in circulating serum LDL cholesterol levels for total population.

	**LDL Cholesterol**						
	**Tertile 1**		**Tertile 2**		**Tertile 3**		
	***n*** **34**		***n*** **35**		***n*** **35**		
	**Mean**	**SD**	**Mean**	**SD**	**Mean**	**SD**	P^*^
LDL cholesterol Δ (%)	−22.41^a^	5.48	−8.81^b^	2.84	7.39^c^	11.46	<0.001
**Gender (%)**
Male	41		29		54		0.092^¥^
Female	59		71		46		
**Baseline characteristics**
Age (years)	60.7	7.5	60.4	7.3	59.8	5.8	0.928
Weight (kg)	79.6	11.9	77.8	16.2	78.1	13.3	0.348
BMI (kg/m^2^)	27.3	3.2	28.3	4.7	27.0	2.6	0.155
Body fat (%)	33.6	8.6	35.7	6.5	31.8	7.3	0.654
Systolic BP (mmHg)^§^	128.1	15.7	127.4	15.8	135.1	23.3	0.317
Diastolic BP (mmHg)^†^	80.5	11.3	83.3	10.6	85.5	14.7	0.294
Total cholesterol (mmol/l)	6.18	1.09	5.83	0.88	5.62	0.85	0.077
HDL-cholesterol (mmol/l)	1.80	0.51	1.81	0.46	1.52	0.47	0.108
LDL-cholesterol (mmol/l)	3.90^a^	0.91	3.46^b^	0.75	3.43^b^	0.74	0.039
TAGs (mmol/l)	1.07^a^	0.41	1.23^a^^b^	0.46	1.49^b^	0.69	0.016
NEFA (mmol/l)	0.58	0.27	0.67	0.32	0.68	0.36	0.368
Glucose (mmol/l)	6.11	0.45	6.31	0.86	6.05	0.42	0.054
Insulin (mU/L)	5.33	2.38	5.86	3.71	5.80	2.48	0.652
hsCRP (mg/L)	1.85	1.65	2.31	1.79	3.04	3.42	0.072

§ *Excludes 3 missing values;*

† *excludes 3 missing values*.

* *Multivariate analysis of baseline characteristics to assess difference between tertiles of responders, with gender and study wave as covariates (P < 0.05), using Bonferroni correction method for multiple comparisons*.

¥ *Chi-squared test was used to assess differences in gender across the tertiles (P < 0.05)*.

abc *Different superscript letters indicate significant differences in mean values across tertiles. n, number; SD, standard deviation; Δ, delta; T1, Tertile 1; T2, Tertile 2; T3, Tertile 3; LDL, low-density lipoprotein; HDL, high-density lipoprotein. BMI, body mass index; BP, blood pressure; LDL, low-density lipoprotein; HDL, high-density lipoprotein; TAGs, triacylglycerols; NEFA, non-esterified fatty acids; hsCRP, high-sensitivity C-reactive protein*.

Multiple regression analysis examining the relationship between the baseline characteristics and serum cholesterol response revealed that the variation in the % change for TC and LDL-c was associated with baseline TC, TAG, body weight and hsCRP (*R*^2^ = 0.23; 0.35; *P* < 0.05, respectively) (Table 4). An additional relationship between HDL-c % change and baseline HDL-c was established, along with sex differences, however this was not as strong (*R*^2^ = 0.11; *P* < 0.05).

**Table 4:** Predictors of total, LDL and HDL cholesterol Δ (%).

**Dependent variable**	**Predictor variable**	**β Coefficient**	**Adjusted *R*^2^**	***P* ^§^**
Total cholesterol Δ (%)				
	Total cholesterol (mmol/l)	−0.401		<0.001
	TAGs (mmol/l)	0.285		0.002
	Weight (kg)	−0.239		0.011
	hsCRP (mg/L)	0.222		0.014
			0.23^*^	<0.001
LDL cholesterol Δ (%)				
	Total cholesterol (mmol/l)	−0.315		<0.001
	TAGs (mmol/l)	0.504		<0.001
	Weight (kg)	−0.189		0.026
	hsCRP (mg/L)	0.239		0.004
			0.36^*^	<0.001
HDL cholesterol Δ (%)				
	HDL cholesterol (mmol/l)	−0.410		<0.001
	Sex	0.231		0.037
			0.11^*^	0.001

§ *Stepwise logistic regression analysis. This model estimates the probability of being a responder*.

* *Adjusted R^2^ selected for the best model. LDL, low-density lipoprotein; HDL, high-density lipoprotein*.

### Clinical response (based on recommended cholesterol cut-off values)

Table 5 compares the frequency of the total population who were above and below the WHO clinical cut-off between baseline and post-intervention. This comparison was also examined across the intervention groups. At baseline, only 16 and 24% of the total population were meeting the recommended TC of <5 mmol/l and LDL-c of <3 mmol/l, respectively. The proportion meeting these increased significantly post-intervention, rising from 16 to 31% for TC and from 24 to 37% for LDL-c (*P* < 0.001). Similar significant increases in those meeting the TC recommendations were observed when split across the intervention groups. Although in Group B, while the proportion meeting the recommendation increased post-intervention, the increase was deemed non-significant (*P* = 0.090). Considering baseline HDL-c levels, the proportion of those meeting the recommended level was high, with 98% of the total population above the recommended HDL-c cut-off (males >1.0 mmol/l; females >1.2 mmol/l). Post-intervention, 97% of the population met the recommendation.

**Table 5:** Comparison of the population meeting the WHO (25) recommended clinical cut-offs between baseline and post-intervention.

	**Baseline**		**Post-intervention**		
	**Not meeting *n* (%)**	**Meeting *n* (%)**	**Not meeting *n* (%)**	**Meeting *n* (%)**	P^*^
**Total cholesterol (<5 mmol/l)**					
Total population	87 (84%)	17 (16%)	72 (69%)	32 (31%)	<0.001
Group A	30 (75%)	10 (25%)	22 (55%)	18 (45%)	<0.001
Group B	33 (92%)	3 (8%)	29 (81%)	7 (19%)	0.090
Group C	24 (86%)	4 (14%)	21 (75%)	7 (25%)	0.002
*P^¥^*		0.138		0.041	
**LDL (<3 mmol/l)**					
Total population	79 (76%)	25 (24%)	66 (64%)	38 (37%)	<0.001
Group A	29 (72%)	11 (28%)	19 (48%)	21 (52%)	<0.001
Group B	28 (78%)	8 (22%)	27 (75%)	9 (25%)	0.001
Group C	23 (82%)	5 (18%)	20 (71%)	8 (29%)	0.015
*P^¥^*		0.642		0.027	
**HDL (males** **>1.0 mmol/l; females** **>1.2 mmol/l)**					
Total population	2 (2%)	102 (98%)	3 (3%)	101 (97%)	<0.001
Group A	1 (2%)	39 (98%)	2 (5%)	38 (95%)	0.050
Group B	1 (3%)	35 (97%)	1 (3%)	35 (97%)	0.028
Group C	0 (0%)	28 (100%)	0 (0%)	28 (100%)	–
*P^¥^*		0.684		0.479	

* *Chi-squared test was used to assess differences within groups (P < 0.05);*

¥ *Chi-squared test was used to assess differences between groups (P < 0.05). n, number; LDL, low-density lipoprotein; HDL, high-density lipoprotein*.

## Discussion

Several studies have examined inter-individual variability in response to the consumption of food and nutrients (9–11, 26–32). The present study is unique in that it is one of the first to examine the factors influencing lipid metabolism response after dairy fat consumption across different cheese matrices as well as examining changes in cholesterol levels from a clinical risk perspective. Here, our data suggests that lipid metabolism response may be influenced by some baseline phenotypic characteristics. Total cholesterol responders, classified here as those who displayed the largest decreases in TC after consuming the dairy fat, had higher baseline levels of TC, HDL-c, and lower levels of TAGs. Similarly, LDL-c responders appeared to be influenced by higher baseline levels of LDL-c and lower TAGs. While our previous work demonstrated a benefit when all of the fat was contained within the cheese (24), this secondary analysis demonstrates that individual variation in response is also influenced by some biochemical factors, regardless of the dietary intervention group.

Inter-individual variation in response to consumption of dietary fat has been reported in TC, LDL-c, and HDL-c (33), with some individuals showing very little change in blood cholesterol, irrespective of significant changes in dietary fat intake (34). This highlights the difficulty in identifying factors that influence response to dietary change (35). Similar to the present study, Kirwan et al. (28) examined phenotypic factors influencing the variation in response to the change in circulating TC levels following dietary intervention. They used data from the Food4Me personalized dietary intervention study and found that while no differences between demographic and anthropometric profiles of responders and non-responders were reported, marked differences between responder and non-responder biochemical phenotypes were observed. Kirwan and colleagues identified a number of differences in baseline fatty acid profiles, specifically, noting higher levels of stearic acid and lower levels of palmitic acid within the responder group (28). In addition, baseline phenotype (demographic, lifestyle, and biochemical profile), characterized by age, alcohol intake, DHA, EPA, eicosenoic acid and *trans-*fatty acid discriminated responders and non-responders in most cases. It is important to note that Kirwan et al. (28) did not distinguish between the sources of *trans*-fat. This is important to consider as evidence has shown that naturally occurring *trans*-fat from natural sources, i.e., ruminant animals, may not have the same adverse effects on diet-related disease risk that industrial sources produced from hydrogenated oils have (36, 37). In line with Kirwan et al., the current analysis also found baseline factors to be an important influence, specifically some biochemical phenotypes (TC responders: higher baseline levels of TC, HDL-c, and lower levels of TAGs; LDL-c responders: higher levels of LDL-c and lower TAGs). While only a small influence in sex was shown, associated with HDL-c % change, other studies have demonstrated significant sex differences in response to lipid consumption. For example, after a 6-month intervention study carried out by Childs et al. (11), significant differences in sex were reported in response to alpha-linoleic acid enriched margarines and spreads. Following the intervention, a greater increase in the eicosapentaenoic acid (EPA) content of plasma phospholipids was observed in females compared to males (11). Furthermore, the current study also demonstrated that the variation in the % change for TC and LDL-c was also influenced by baseline TC, TAG, body weight and hsCRP. As previously mentioned, TC and LDL-c responders who displayed the largest decreases in TC and LDL-c, respectively, had higher baseline concentrations. This suggests that the reductions observed could potentially be due to higher baseline levels providing more room for reduction. However, while the group was split into tertiles based on their % change and ranked from the largest decrease to the largest increase, the mean baseline concentrations for TC and LDL-c were in fact above the recommended lipid levels across all three tertiles. This suggests that the response being driven by baseline concentrations is not simply an artifact of a higher starting concentration. Although investigating the effects of an anti-inflammatory nutrition supplement and not specific to lipid consumption, McMorrow et al. (30), observed a significant variation in response within a cohort of overweight adolescents. They reported that the supplement modulated adiponectin biology, an early predictor of type 2 diabetes risk. In addition, improvements in insulin resistance were observed among a sub-cohort considered to be responders. Interestingly, responders exhibited insulin resistance and dyslipidemic phenotypes with higher homeostatic model assessment for insulin resistance and β-cell function (HOMA-IR and HOMA- β, respectively), TC, LDL-c, and lower quantitative insulin sensitivity check index (QUICKI) at baseline compared to non-responders (30). No differences between responders and non-responders were reported across demographic characteristics such as sex, age, BMI, and body composition. However, this could be due to their selective cohort of overweight adolescents. The demographic findings presented in the current study were similar, apart from a small influence in sex associated with HDL-c % change.

Although not examined in this present analysis, it is important to consider the influence of genetic variation in response to dietary intervention as this could be useful when tailoring dietary advice. A study by Shatwan et al. (12) considered the impact of genetic variation in response to lipid consumption and investigated a potential diet-gene interaction in individuals with a moderate risk of CVD. After a 16-week intervention of diets high in SFA, MUFA or *n-*6 PUFA, they reported that only TT homozygotes showed a significant reduction in TC after the MUFA diet compared to the SFA or n-6 PUFA diets when stratified for apolipoprotein E (APOE) SNP rs1064725. Furthermore, Chouinard-Watkins and colleagues (13) examined changes in circulating lipid profiles in a healthy cohort after an 8-week intervention consuming a high SFA diet, with the addition of docosahexaenoic acid (DHA) and EPA. When stratified by body mass index (BMI; low <25, high >25), they discovered that APOE4 carriers were lower responders to DHA supplementation compared with non-carriers of the allele, although, this was only evident in the high-BMI group (13).

While an individual's phenotype are important factors to consider when examining variation in response, the influence of the food matrix and the food source also needs to be considered, further adding to the already complex nature of inter-individual variation. The focus on whole foods as opposed to individual nutrients is increasing as research has demonstrated that a food product is more than the sum of its individual components (38). Evidence shows this is particularly true for dairy products (21, 24, 38–41). With a range of complex physical and nutritional structures, the dairy matrix can influence the digestion, absorption and bioactive nature of the nutrients it holds, as well as their biological effects (38). Focusing specifically on fat derived from the dairy food group, several studies have concluded that fat when contained within the dairy matrix can result in beneficial effects on cardiovascular health (21, 39–41). A meta-analysis by Drouin-Chartier et al. (20) examined the impact of dairy consumption and dairy fat on cardiometabolic disease risk factors and concluded that when SFA is consumed within the dairy matrix, it may have favorable or neutral associations with cardiometabolic health. This has been particularly evident in cheese (24). Despite the fact that cheese consumption is a significant contributor of SFA intake in many Western diets, the overarching evidence does not associate cheese with diseases such as CVD or stroke (42, 43). In accordance with our previous intervention (24), a number of clinical studies have reported cholesterol-lowering effects when dairy fat is consumed in the form of cheese compared with butter (44, 45), suggesting that the cheese matrix can modulate the effect of dairy fat on cardiovascular health (46). Furthermore, Alexander et al. (21), carried out a meta-analysis of prospective cohort studies examining dairy intake and CVD. While they concluded that there may be associations between dairy consumption and a reduction in CVD risk, additional data are needed to further examine potential dose–response patterns (21). Recently, new evidence has emerged demonstrating that the cheese matrix can modulate dairy fat digestion (46). Drouin-Chartier and colleagues compared the impact of cheeses with different hardnesses (firm cheddar, soft cream cheese, and butter) on post-prandial response and found that when the dairy fat is contained within the soft cream cheese matrix it is digested more rapidly than when provided in the butter or cheddar matrix (46). However, another meta-analysis by Drouin-Chartier et al. (22) investigated the association of changes in dairy product consumption with subsequent risk of type 2 diabetes in US men and women. Conversely, they found that an increase in yogurt consumption was associated with a moderately lower risk of T2D, whereas increasing cheese consumption was associated with a higher risk (22).

Previously, we reported a matrix effect in response to a 6-week dairy fat intervention, whereby the greatest cholesterol reductions were observed when dairy fat was eaten within the matrix of cheese. Here, we find individual variation in response to that intervention, across all of the food matrices in those dietary intervention groups (24). In this analysis, we have uniquely examined both the inter-individual variation in response, and variation according to the food form, where factors influencing response were considered at a total cohort level and across each intervention group. While the cheese matrix has been shown to influence lipid metabolism, within this secondary analysis, we find that baseline biochemical markers and phenotype also appear to influence response, irrespective of food form. Nevertheless, if we consider the distribution within tertiles of % change of TC, there was a greater proportion of individuals within tertile 1 (responders) who consumed full-fat cheese (40%), compared to those who consumed butter (18%). Thus, demonstrating that more people who consumed full-fat cheese were considered responders than other intervention groups. In terms of clinical relevance, the percentage of the population meeting the recommended TC and LDL-c levels nearly doubled following the intervention, increasing from 16 to 31%, and 24 to 37%, respectively. This change was also greatest in those who consumed full-fat cheese, indicating that cholesterol in terms of clinical risk has the potential to improve when individuals consume dietary fat within the cheese matrix.

Strengths of this study include its robust RCT study design, the number of phenotypic variables included in the analyses, and the application of these to consider potential effects on clinical risk factors. However, there are several limitations that should be acknowledged. Across the diet groups, Group C experienced a higher dropout rate. This was most likely due to the lower palatability of study foods compared to the other cheese groups. Further, responders and non-responders were classified based on the % change on cholesterol level from baseline to post-intervention, with responders displaying the highest change, and non-responders displaying the lowest change, and these two extremes of response were compared. It must be acknowledged that this method of response categorization is somewhat arbitrary. This was seen in the clinical response to cholesterol risk, which was based on the direction of the clinical change, post-intervention. A small number (*n* = 5) of individuals responded negatively from a clinical risk perspective, i.e., moved from a “healthy” category into a higher risk category, but were classified as non-responders from our categorization method. Albeit arbitrary, it is important that different methods categorizing responders are considered, to ensure that the most appropriate method can be identified when influencing dietary advice relevant to healthcare professionals and consumers.

With the growing number of publications investigating inter-individual response to the consumption of foods and nutrients, it is essential to harness this information and begin to develop approaches and strategies to tailor nutritional recommendations for individual groups (6). Utilizing metabolic profiles to identify differing responses to dietary interventions can aid the development of personalized nutrition approaches, thus reducing diet-related disease risk. In this analysis, we demonstrate that baseline phenotypic characteristics (namely TC, LDL-c, and TAGs levels), may influence lipid metabolism response in overweight individuals following the consumption of dairy fat, when all diet groups are considered together as one dairy fat intervention. However, demographic, and anthropometric characteristics did not influence response. Although dairy fat within the cheese matrix has shown beneficial effects on cholesterol levels, individual characteristics play an important role in inter-individual variance, and biochemical factors have shown to influence lipid metabolism response within this cohort. These results are highly relevant to the current topic of tailored nutrition-related advice, which can support the detection of early-onset diet-related diseases. However, additional work is needed to further examine and understand the complexities of inter-individual variation in order to advance the development of precision nutrition and influence change in dietary recommendations.

## Data availability statement

The datasets presented in this article are not readily available because the archived data will be stored electronically in a password protected file on a secure drive on a password protected, encrypted computer in the UCD Institute for Food and Health which is only accessible to study investigators in line with current HREC data storage and retention guidelines. This data will not be accessible to others. Requests to access the datasets should be directed to Prof. EG, eileen.gibney@ucd.ie.

## Ethics statement

The studies involving human participants were reviewed and approved by the Human Ethics Research Committee of University College Dublin. The patients/participants provided their written informed consent to participate in this study.

## Author contributions

EG and EF were principal investigators, designed the study, provided valuable knowledge, and scientific consultation throughout the study. NN contributed to the study design. AO'C and NB performed the statistical analyses. AO'C, EG, and EF analyzed and interpreted the data and wrote the manuscript. All authors read and approved the final version of the draft before publication.

## Funding

This study was funded by Food for Health Ireland, a dairy technology center part-financed by Enterprise Ireland and partly by dairy companies in Ireland. While industry-affiliated partners were invited to comment on the initial study design, the researchers made the final decisions (grant numbers TC-2013-001 and TC-2018-0025).

## Conflict of interest

Authors EG, AO'C, and EF have previously received travel expenses and/or speaking honoraria from the National Dairy Council, UK, USA and Norway. Authors EG, AO'C, and EF have received research funding through the Food for Health Ireland project, funded by Enterprise Ireland, grant numbers TC-2013-001 and TC20180025. The remaining authors declare that the research was conducted in the absence of any commercial or financial relationships that could be construed as a potential conflict of interest.

## Publisher's note

All claims expressed in this article are solely those of the authors and do not necessarily represent those of their affiliated organizations, or those of the publisher, the editors and the reviewers. Any product that may be evaluated in this article, or claim that may be made by its manufacturer, is not guaranteed or endorsed by the publisher.
