# Supplementary material for: Determination of factors associated with serum cholesterol response to dairy fat consumption in overweight adults: Secondary analysis from an RCT
Source: Front Nutr. 2022 Aug 3;9:945723. doi: 10.3389/fnut.2022.945723 (PMC9382121; doi:10.3389/fnut.2022.945723)
Supplement: Supplementary file 1 [file Data_Sheet_1.docx]

| **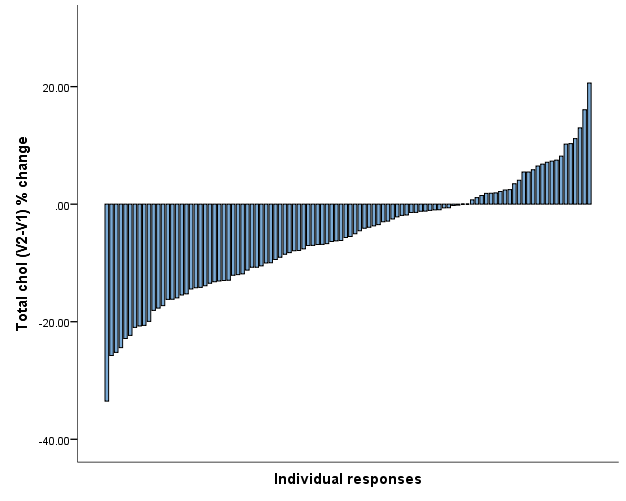 Total population** | **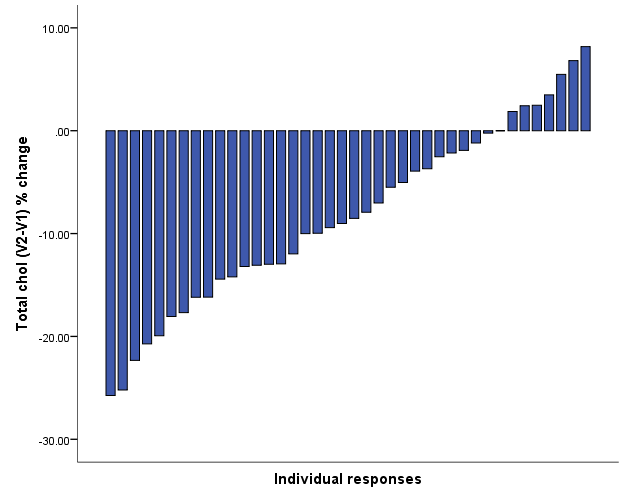Group A** |
| --- | --- |
| 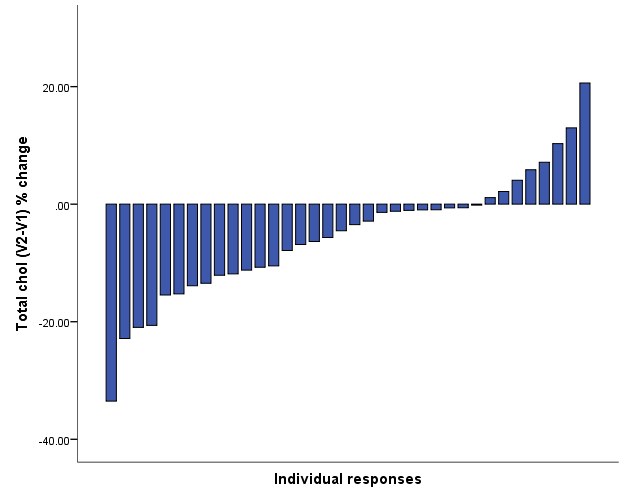**Group B** | 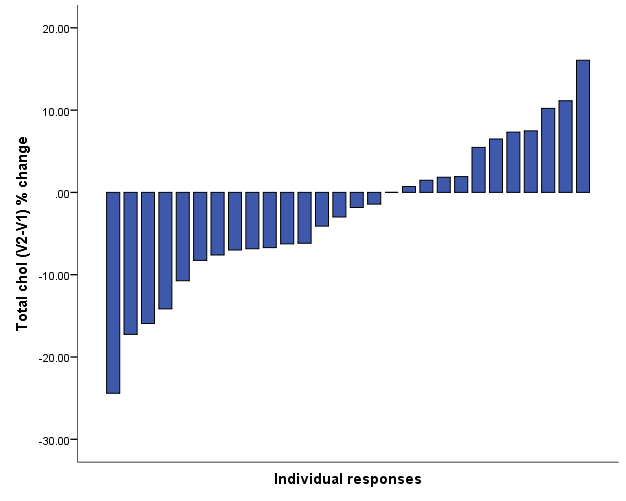**Group C** |

| **Total population**  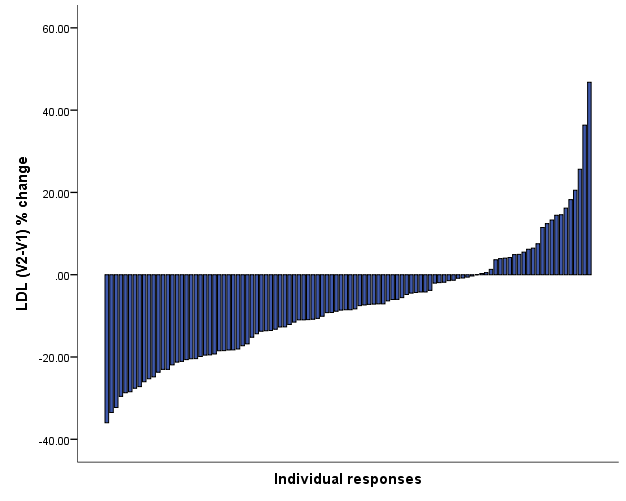 | 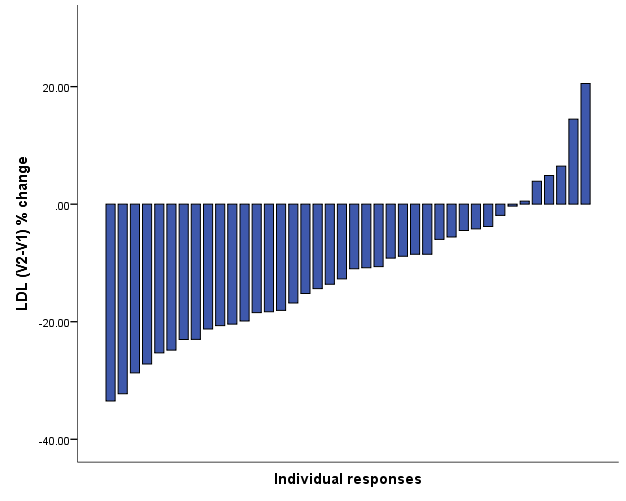**Group A** |
| --- | --- |
| 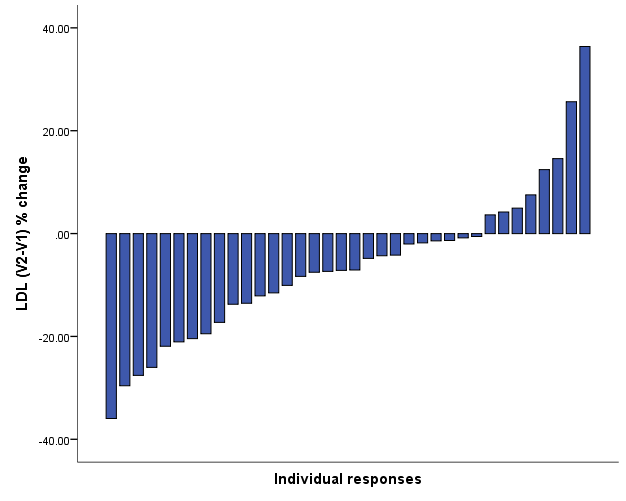**Group B** | 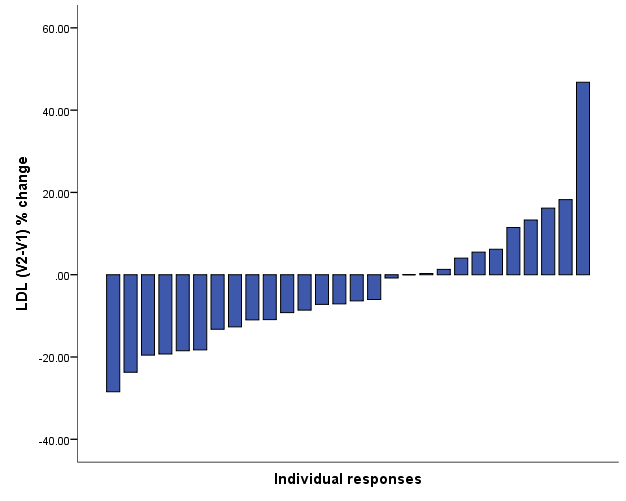**Group C** |

| 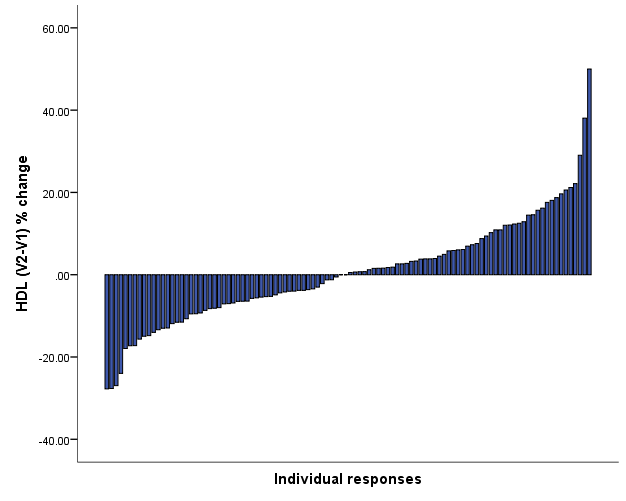 **Total population** | 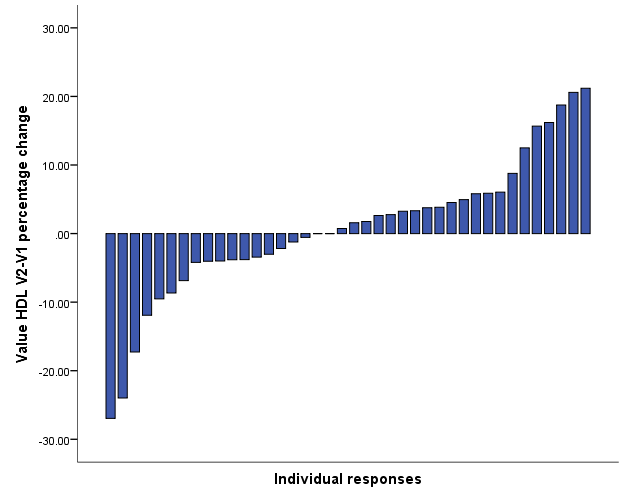**Group A** |
| --- | --- |
| 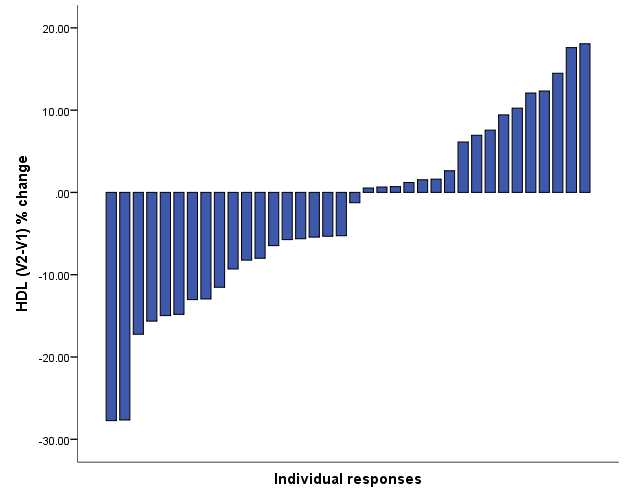**Group B** | 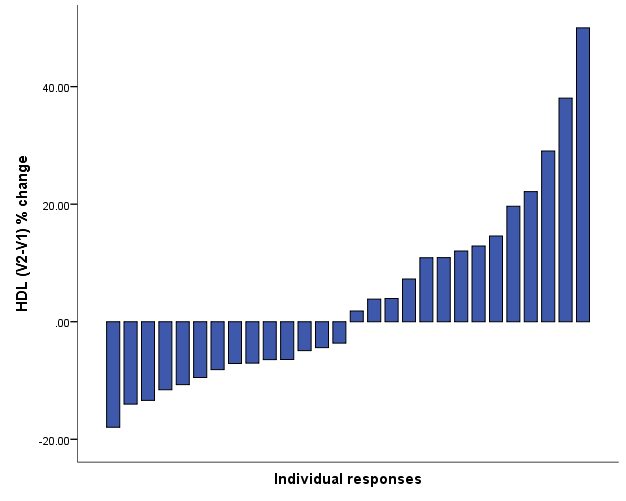**Group C** |
