# Supplementary material for: Determination of factors associated with serum cholesterol response to dairy fat consumption in overweight adults: Secondary analysis from an RCT
Source: Front Nutr. 2022 Aug 3;9:945723. doi: 10.3389/fnut.2022.945723 (PMC9382121; doi:10.3389/fnut.2022.945723)
Supplement: Supplementary file 2 [file Data_Sheet_2.docx]

**Supplementary Tables**

| **Supplementary Table 1.** Nutritional composition of the intervention diets | | | | | | |
| --- | --- | --- | --- | --- | --- | --- |
| Diet | Intervention | Energy | Protein | Fat | Lactose | Calcium |
|  |  | kcal/d | g/d | g/d | g/d | mg/d |
| A | 120 g full-fat Irish cheddar cheese | 481.2 | 31.2 | 40.8 | 0.12 | 828 |
| B | 120 g reduced-fat Irish cheddar cheese + 21 g butter | 517.0 | 30.0 | 43.2 | 0.12 | 900 |
| C | 49 g butter + 30 g calcium caseinate + CaCO_3_ supplement | 476.2 | 27.3 | 39.2 | 0.01 | 817 |
| D | 120 g full-fat Irish cheddar cheese | 481.2 | 31.2 | 40.8 | 0.12 | 828 |
| Adapted from Feeney et al.^(21)^ | |  |  |  |  |  |

| **Supplementary Table 2a.** Predictors of total, LDL and HDL cholesterol Δ (%) for Group A^¥^ | | | | |
| --- | --- | --- | --- | --- |
| Dependent Variable | Predictor variable | *β* Coefficient | Adjusted R^2^ | *P*^§^ |
| ***Total cholesterol Δ (%)*** |  |  |  |  |
|  | LDL cholesterol (mmol/l) | -0.567 |  | <0.001 |
|  | hsCRP (mg/L) | 0.375 |  | 0.005 |
|  |  |  | 0.39* | <0.001 |
| ***LDL cholesterol Δ (%)*** |  |  |  |  |
|  | TAGs (mmol/l) | 0.726 |  | <0.001 |
|  | LDL cholesterol (mmol/l) | -0.548 |  | <0.001 |
|  | hsCRP (mg/L) | 0.383 |  | <0.001 |
|  | BMI (kg/m²) | -0.259 |  | 0.012 |
|  | NEFA (mmol/l) | -0.261 |  | 0.027 |
|  | Glucose (mmol/l) | -0.202 |  | 0.045 |
|  |  |  |  |  |
|  |  |  | 0.67* | <0.001 |
| ***HDL cholesterol Δ (%)*** |  |  |  |  |
|  | TAGs (mmol/l) | 0.433 |  | 0.004 |
|  | BMI (kg/m²) | -0.412 |  | 0.006 |
|  | Total Cholesterol (mmol/l) | -0.421 |  | 0.004 |
|  |  |  | 0.30* | 0.001 |
| ^¥^full-fat cheddar cheese (*n*=40). ^§^Stepwise logistic regression analysis. This model estimates the probability of being a responder. *Adjusted R² selected for the best model. LDL, low-density lipoprotein; HDL, high-density lipoprotein. | | | | |

| **Supplementary Table 2b.** Predictors of total, LDL and HDL cholesterol Δ (%) for Group B^¥^ | | | | |
| --- | --- | --- | --- | --- |
| Dependent Variable | Predictor variable | *β* Coefficient | Adjusted R^2^ | *P*^§^ |
| ***Total cholesterol Δ (%)*** |  |  |  |  |
|  | LDL cholesterol (mmol/l) | 0.415 |  | 0.010 |
|  | hsCRP (mg/L) | -0.0325 |  | 0.038 |
|  |  |  | 0.23* | 0.006 |
| ***LDL cholesterol Δ (%)*** |  |  |  |  |
|  | TAGs (mmol/l) | 0.488 |  | 0.003 |
|  |  |  | 0.22* | 0.003 |
| ***HDL cholesterol Δ (%)*** |  |  |  |  |
|  | HDL cholesterol (mmol/l) | -0.360 |  | 0.034 |
|  |  |  | 0.10* | 0.034 |
| ^¥^reduced-fat cheese plus butter (*n*=36). ^§^Stepwise logistic regression analysis. This model estimates the probability of being a responder. *Adjusted R² selected for the best model. LDL, low-density lipoprotein; HDL, high-density lipoprotein. | | | | |

| **Supplementary Table 2c.** Predictors of total, LDL and HDL cholesterol Δ (%) for Group C^¥^ | | | | |
| --- | --- | --- | --- | --- |
| Dependent Variable | Predictor variable | *β* Coefficient | Adjusted R^2^ | *P*^§^ |
| ***LDL cholesterol Δ (%)*** |  |  |  |  |
|  | BP Systolic (mmHg) | 0.679 |  | <0.001 |
|  |  |  | 0.44* | <0.001 |
| ***HDL cholesterol Δ (%)*** |  |  |  |  |
|  | NEFA (mmol/l) | 0.458 |  | 0.019 |
|  |  |  | 0.18* | 0.019 |
| ^¥^Butter, calcium caseinate powder, and a calcium supplement (CaCO3; *n*=28). ^§^Stepwise logistic regression analysis. This model estimates the probability of being a responder. *Adjusted R² selected for the best model. LDL, low-density lipoprotein; HDL, high-density lipoprotein.  A model to establish the predictors of total cholesterol Δ (%) could not be created in Group C as the variables entered did not explain the variation. The lower number of participants within Group C (*n*=28) may have contributed to this. | | | | |

| **Supplementary Table 3.** Percentage change in total cholesterol, LDL and HDL across tertiles of percentage change derived from population level, and within each intervention group (A-C) | | | | | | | | | | | | | |
| --- | --- | --- | --- | --- | --- | --- | --- | --- | --- | --- | --- | --- | --- |
|  | T1 | | |  | T2 | | |  | T3 | | |  | *P** |
|  | *n* | Mean | SD |  | *n* | Mean | SD |  | *n* | Mean | SD |  |  |
| ***Total cholesterol Δ (%)*** |  |  |  |  |  |  |  |  |  |  |  |  |  |
| Total population | 34 | -16.76^a^ | 5.31 |  | 35 | -5.35^b^ | 2.73 |  | 35 | 4.59^c^ | 5.21 |  | <0.001 |
|  |  |  |  |  |  |  |  |  |  |  |  |  |  |
| Group A | 13 | -18.23^a^ | 4.30 |  | 14 | -8.43^b1^ | 3.10 |  | 13 | 1.75^c^ | 3.51 |  | <0.001 |
| Group B | 12 | -16.82^a^ | 6.65 |  | 12 | -4.39^b2^ | 3.11 |  | 12 | 5.16^c^ | 6.67 |  | <0.001 |
| Group C | 9 | -12.47^a^ | 5.98 |  | 10 | -2.73^b2^ | 3.01 |  | 9 | 7.55^c^ | 4.50 |  | <0.001 |
| *P*^¥^ |  | 0.070 |  |  |  | <0.001 |  |  |  | 0.071 |  |  |  |
| ***LDL Δ (%)*** |  |  |  |  |  |  |  |  |  |  |  |  |  |
| Total population | 34 | -22.41^a^ | 5.48 |  | 35 | -8.70^b^ | 2.88 |  | 35 | 7.67^c^ | 11.27 |  | <0.001 |
|  |  |  |  |  |  |  |  |  |  |  |  |  |  |
| Group A | 13 | -24.49^a^ | 4.76 |  | 14 | -12.61^b1^ | 3.51 |  | 13 | 1.88^c^ | 8.11 |  | <0.001 |
| Group B | 12 | -21.56^a^ | 7.18 |  | 12 | -6.35^b2^ | 2.99 |  | 12 | 8.76^c^ | 11.84 |  | <0.001 |
| Group C | 9 | -18.29^a^ | 5.54 |  | 10 | -5.59^b2^ | 4.01 |  | 9 | 13.66^c^ | 13.68 |  | <0.001 |
| *P*^¥^ |  | 0.063 |  |  |  | <0.001 |  |  |  | 0.100 |  |  |  |
| ***HDL Δ (%)*** |  |  |  |  |  |  |  |  |  |  |  |  |  |
| Total population | 34 | -12.41^a^ | 6.40 |  | 36 | -0.47^b^ | 3.01 |  | 34 | 14.33^c^ | 9.71 |  | <0.001 |
|  |  |  |  |  |  |  |  |  |  |  |  |  |  |
| Group A | 13 | -9.88^a^ | 8.04 |  | 14 | 0.92^b^ | 2.16 |  | 13 | 11.13^c1^ | 6.58 |  | <0.001 |
| Group B | 12 | -15.09^a^ | 6.57 |  | 12 | -2.54^b^ | 3.32 |  | 12 | 9.93^c1^ | 5.29 |  | <0.001 |
| Group C | 9 | -11.04^a^ | 3.62 |  | 10 | 0.20^a^ | 6.19 |  | 9 | 23.25^b2^ | 13.43 |  | <0.001 |
| *P*^¥^ |  | 0.185 |  |  |  | 0.151 |  |  |  | 0.005 |  |  |  |
| *Univariate analysis of baseline characteristics to assess differences between tertiles of percentage change, with gender and study wave as covariates (*P*<0.05). ^¥^Univariate analysis of baseline characteristics to assess differences between intervention groups A-C, with gender and study wave as covariates (*P*<0.05). Both used Bonferroni correction method for multiple comparisons. ^abc^Different superscript letters indicate significant differences in mean values across tertiles. ^123^Different superscript letters indicate significant differences in mean values across groups. *n*, number; SD, standard deviation; Δ, delta; T1, Tertile 1; T2, Tertile 2; T3, Tertile 3; LDL, low-density lipoprotein; HDL, high-density lipoprotein. | | | | | | | | | | | | | |

| **Supplementary Table 4.** Comparison of baseline characteristics across tertiles of percentage change in circulating serum HDL cholesterol levels for total population | | | | | | | |
| --- | --- | --- | --- | --- | --- | --- | --- |
|  | HDL Cholesterol | | | | | |  |
|  | Tertile 1 | | Tertile 2 | | Tertile 3 | |  |
|  | *n* 34 | | *n* 36 | | *n* 34 | |  |
|  | Mean | SD | Mean | SD | Mean | SD | *P** |
| HDL cholesterol Δ (%) | -12.33^a^ | 6.48 | -0.47^b^ | 3.01 | 14.08^c^ | 9.92 | <0.001 |
|  |  |  |  |  |  |  |  |
| ***Gender (%)*** |  |  |  |  |  |  |  |
| Male | 50 |  | 42 |  | 32 |  | 0.335 |
| Female | 50 |  | 58 |  | 68 |  |  |
| ***Baseline Characteristics*** | |  |  |  |  |  |  |
| Age (years) | 59.70 | 7.23 | 61.42 | 6.63 | 59.69 | 6.70 | 0.481 |
| Weight (kg) | 81.85 | 12.67 | 76.71 | 12.95 | 77.11 | 15.51 | 0.422 |
| BMI (kg/m^2^) | 28.05 | 3.84 | 27.43 | 2.52 | 27.23 | 4.38 | 0.802 |
| Body fat (%) | 32.87 | 8.56 | 33.77 | 7.13 | 34.51 | 7.23 | 0.865 |
| Systolic BP (mmHg) ^§^ | **125.97^a^** | **14.96** | **137.00^b^** | **22.09** | **126.63^ab^** | **16.09** | **0.016** |
| Diastolic BP (mmHg) ^†^ | 80.88 | 8.67 | 85.40 | 15.18 | 82.72 | 11.96 | 0.207 |
| Total cholesterol (mmol/l) | 6.12^a^ | 0.97 | 5.95^ab^ | 1.02 | 5.56^b^ | 0.83 | 0.018 |
| HDL-cholesterol (mmol/l) | **1.86^a^** | **0.53** | **1.71^ab^** | **0.49** | **1.56^b^** | **0.42** | **0.001** |
| LDL-cholesterol (mmol/l) | 3.75 | 0.84 | 3.63 | 0.87 | 3.40 | 0.75 | 0.247 |
| TAGs (mmol/l) | 1.13 | 0.44 | 1.35 | 0.65 | 1.31 | 0.53 | 0.115 |
| NEFA (mmol/l) | 0.56 | 0.26 | 0.70 | 0.40 | 0.66 | 0.26 | 0.219 |
| Glucose (mmol/l) | 6.10 | 0.69 | 6.18 | 0.59 | 6.19 | 0.57 | 0.581 |
| Insulin (mU/L) | 4.96 | 2.34 | 6.05 | 2.73 | 5.95 | 3.52 | 0.265 |
| hsCRP (mg/L) | 2.56 | 2.05 | 2.66 | 3.12 | 1.91 | 1.86 | 0.261 |
| ^§^excludes 3 missing values; ^†^excludes 3 missing values. *Multivariate analysis of baseline characteristics to assess difference between tertiles of responders, with gender and study wave as covariates (*P*<0.05), using Bonferroni correction method for multiple comparisons. ^¥^Chi-squared test was used to assess differences in gender across the tertiles (*P*<0.05). ^abc^Different superscript letters indicate significant differences in mean values across tertiles. *n,* number; SD, standard deviation; Δ, delta; T1, Tertile 1; T2, Tertile 2; T3, Tertile 3; LDL, low-density lipoprotein; HDL, high-density lipoprotein. BMI, body mass index; BP, blood pressure; LDL, low-density lipoprotein; HDL, high-density lipoprotein; TAGs, triacylglycerols; NEFA, non-esterified fatty acids; hsCRP, high-sensitivity C-reactive protein. | | | | | | | |
